# Bariatric and metabolic surgery in Asia: Where are we, and where are we going?

**DOI:** 10.1111/jdi.12893

**Published:** 2018-08-03

**Authors:** Kazuki Yasuda

**Affiliations:** ^1^ Department of Metabolic Disorder Diabetes Research Center National Center for Global Health and Medicine Tokyo Japan

The worldwide prevalence of obesity keeps increasing, especially in Asian and African countries, but pharmacological treatment of obesity remains a huge challenge. Surgical intervention, known as bariatric surgery, achieves dramatic and sustained bodyweight loss in morbidly obese individuals; concomitantly, it results in substantial improvement of obesity‐related comorbidities, especially diabetes mellitus, as many patients with diabetes can reach “complete remission” (glycated hemoglobin <6% without medication). Based on this observation, the term “metabolic surgery” was generated. Clinical trials have reported additional beneficial impacts on other disorders, such as hypertension, hyperlipidemia, non‐alcoholic fatty liver disease, cardiovascular events and certain kinds of cancer. Some studies have also reported improvement in quality of life and economic benefits.

Recently, several large‐scale randomized clinical trials (RCTs) comparing surgical and non‐surgical (medical) treatment of obese patients with diabetes have been published^1^; these trials reproducibly showed far greater weight loss and better glycemic control in surgery groups for at least 5 years. Interestingly, metabolic improvement was achieved in patients with diabetes with both morbid and “non‐morbid” obesity.

Based on these reports, as well as intensive discussion in the third World Congress on Interventional Therapies for Type 2 Diabetes and the second Diabetes Surgery Summit held in 2015 in London, the American Diabetes Association recently announced an algorithm to guide surgical treatment for obese patients with type 2 diabetes^2^; diabetes societies from many countries, including Asian nations, are largely in agreement with it. Thus, surgical treatment is now established as a first‐line therapy for diabetes patients with morbid obesity, and as a reasonable choice for diabetes patients with non‐morbid obesity.

From 29 to 31 March 2018, the Congress of Asia Pacific Metabolic and Bariatric Surgery Society (APMBSS) was held in Tokyo, Japan, by the congress president, Dr Kazunori Kasama. Although the majority of the participants were surgeons or surgical staff members, a variety of professionals, including physicians, dietitians, psychologists, mental and physical therapists, and social coordinators, also joined this fantastic meeting with beautiful cherry blossoms around the hotel. As a physician, I was deeply impressed by the tight Pan‐Asian unity of those engaged in surgical interventions for obesity and metabolic disorders, and would like to share with the readers some of the topics discussed at the congress.

## Bariatric/Metabolic Surgery in Asia

It is often said that the pathophysiology of type 2 diabetes mellitus is considerably different between the West and the East; whereas obesity and insulin resistance are the major predisposing factors in white people, impaired insulin secretion is the major predisposing factor among Asians.

Although many reports on bariatric/metabolic surgery in morbidly obese patients have been published with a focus on white populations, it remains rather controversial whether equivalent effects can be obtained in Asian patients.

In Asia, bariatric surgery was first carried out in 1974 in Taiwan^3^; with the rapidly increasing number of obese individuals, surgical intervention in cases of obesity and/or diabetes is becoming increasingly popular, although the number of operations significantly varies among countries.

In the congress, Asian experiences of bariatric/metabolic surgery, both nationwide surveys and RCTs, were presented from many countries, including Japan^4^. There are considerable variations in the body mass index (BMI) inclusion criteria, modes of operation, follow‐up time and primary end‐points (reduction in excess bodyweight, improvement of diabetes or other comorbidities), but most of the studies showed that Asian patients responded well to surgery with a minimum number of adverse events. This is not surprising, as the main pathophysiology of diabetes might not differ widely between white people and Asian people; for example, recent genome‐wide association studies showed that most of the susceptibility single‐nucleotide polymorphisms for type 2 diabetes mellitus are shared among ethnic groups.

It is also well‐known that Asians are more prone to diabetes mellitus than white people with the same degree of BMI. A current notion is that a large portion of the metabolic effects of bariatric/metabolic surgery can be explained by bodyweight‐independent pathways (as discussed below). Interestingly, in the aforementioned algorithm^2^, BMI criteria for Asians were set at lower levels by 2.5 kg/m^2^ in each category; surgery is highly recommended for diabetes patients with BMI >37.5 kg/m^2^, rather than >40 kg/m^2^, and should be considered as a candidate therapy for Asians with BMI of 32.5–37.5 kg/m^2^, or even those with BMI of 27.5–32.5 kg/m^2^ if their diabetes is poorly controlled. Notably, this means that there are more potential candidates for bariatric/metabolic surgery among Asians than previously expected.

## Mechanisms of Bariatric/Metabolic Surgery

Previously, bariatric/metabolic surgery was simply thought of as a mechanical intervention to modify food intake and absorption. However, it is now established that this surgery exerts a series of “physiological” responses, which serve to reduce bodyweight and improve metabolism, including glucose homeostasis.

**Figure 1:**
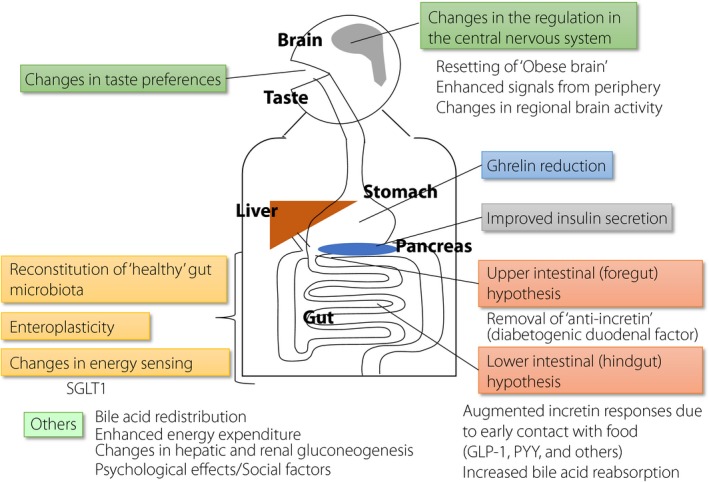
Mechanisms of bodyweight‐independent metabolic effects of bariatric/metabolic surgery. Not only the gastrointestinal tract, but many other organs, such as brain, pancreas and liver, also make a great contribution. Note that no single biological process can explain the whole picture. GLP‐1, glucagon‐like peptide‐1; PYY, peptide YY; SGLT1, sodium–glucose cotransporter 1.

Guest lecturers Professor Lee Kaplan and Professor David Cummings gave exciting overviews of the many biological processes affected by surgical intervention (Figure 1). Proposed mechanisms include reduction of the appetite‐inducing hormone ghrelin, upper gut (foregut) effects (the reduction of “anti‐incretin” from the duodenum), lower gut (hindgut) effects (early contact of food with the lower gut, which increases incretins), redistribution of bile acids (which accelerates energy expenditure), reconstitution of a “healthy” gut microbiota, changes of the energy set‐point in the brain, changes in taste preference and improved insulin secretion. None of these can function as a single major factor, and a complex combination of these factors seems important for each patient. Nevertheless, there likely remain unknown factors or pathways mediating the dramatic effects of surgery.

## Indication of Patients for Surgical Therapy

For successful bariatric/metabolic surgery, it is important for physicians to select the right patients at the right time of the disease course for the surgery. However, it remains difficult to definitively predict which patients will adequately respond to the surgery.

It is generally assumed that younger patients who have had a shorter duration of diabetes, in whom insulin secretory capacity is expected to be preserved, are likely to respond better, but there have been inconsistencies among studies.

Several scoring systems have been proposed to predict the responsiveness of patients to surgical treatment; one of the most well‐known is the “ABCD” score, developed by Professor WJ Lee and colleagues in Taiwan^5^, which consists of A (Age), B (BMI), C (C‐peptide) and D (Diabetes duration). This scoring method has proven to be practically very useful. However, it is not yet known whether the same prediction score can be applied to weight loss and diabetic remission, or to different modes of operations.

## Comparison of the Modes of Operation and Alternative Non‐Surgical Devices

Today, the two major modes of operation are sleeve gastrectomy (SG) and Roux‐en‐Y gastric bypass; there have been many trials, both RCTs and non‐RCTs, to compare the efficacy and safety between these two. In most reports, SG showed satisfactory effects with fewer adverse events, but some reports showed better metabolic improvements with bypass operations.

The original Roux‐en‐Y gastric bypass procedure is not suitable in Asian populations because of their relatively high incidence of gastric cancer; thus, laparoscopic SG with duodenal‐jejunal bypass was invented by Dr Kasama, the president of the congress. A national report from Japan showed that laparoscopic SG with duodenal‐jejunal bypass might be more efficient than SG for obese patients with inadequately controlled diabetes^6^.

Other modified procedures have been proposed and were discussed in the congress. Although some seemed to be similarly effective, at least for 1 year, most were still in the preliminary phase; it is not within the scope of this Editorial to review those newer procedures.

We physicians are excited to imagine the possibility that the beneficial effects of surgical treatment could be achieved by non‐surgical devices. Several types of endoscopic bariatric therapies have been developed, including endoscopic intragastric balloon placement and its derivative, the adjustable gastric balloon, as well as endoscopic duodenal‐jejunal bypass sleeve therapy. Although a prototype, EndoBarrier, based on the “upper gut hypothesis” was discontinued due to reported cases of liver abscess, other devices have been approved by the US Food and Drug Administration. There are other promising products and trials, such as a combination of endoscopic bariatric therapies with IT (information technology) systems.

## Importance of Bariatric Physicians

As morbidly obese patients generally have a variety of serious medical, mental and social problems, multidisciplinary care is essential for their management. This is also the case with pre‐and post‐surgical care, and physicians are expected to play a central role. Such physicians have recently been described as “bariatric physicians,” and their unprecedented roles were discussed in a specialized session. Clearly, they should show an in‐depth understanding of bariatric/metabolic surgery, and should serve to orient and organize the medical team and patients. I also propose that there is a “mindset” required for bariatric physicians. We physicians tend to insist that obesity or diabetes should be managed non‐invasively and exclusively by non‐surgeons; thus, we are very often reluctant to send our obese/diabetic patients to undergo bariatric surgery. However, by managing our patients on our own for too long (i.e., until their insulin secretory capacity becomes exhausted), we might miss the best time for surgery. Bariatric physicians should try to advance patients to surgery at the right time; the key for this would be mutual understanding and respect between physicians and surgeons.

## Issues to be Solved

There remain a number of issues to be addressed. First, the effects of bariatric surgery on non‐morbidly obese patients are of great interest. This is especially important in Asian people, who develop comorbidities at relatively lower BMI than white people; multiple trials, including RCTs, have been carried out to determine these effects. In non‐morbidly obese patients, management of diabetes (rather than obesity itself) would be the main goal of surgery. In Japan, just 400–500 patients per year are undergoing this treatment; however, we recently published two papers showing the efficacy and relative safety of bariatric/metabolic surgery in treatment of non‐morbidly obese Japanese patients with medically intractable diabetes mellitus^7^, ^8^.

Second, the mechanisms underlying the effects of bariatric surgery should be more precisely elucidated. If the key molecules or pathways are discovered, we will then have novel therapeutic targets that might be affected by non‐surgical methods, including new drugs. We can also explore biomarkers that show the efficacy of surgery.

Weight regain and diabetes relapse, one or more years after operation, are serious issues that remain during long‐term follow up of post‐surgical patients. The detailed pathophysiology underlying these phenomena has not yet been elucidated and gold standard treatment has not been established, but Professor Kaplan greatly emphasized the importance of medical care and lifestyle interventions.

Long‐term safety issues are of great importance, especially for non‐surgeons. After bariatric/metabolic surgery, patients should be carefully monitored from a nutritional point of view. Recent reports have shown a considerable increase in some complications after operation, such as reoperation, psychiatric disorders and nutritional problems, including iron‐deficient anemia, micronutrient deficiency and osteoporosis.

Many unanswered questions remain in this field, all of which are quite important and interesting. I believe that bariatric/metabolic surgery in Asia will provide a novel perspective regarding the pathophysiology and management of diabetes and obesity in the near future.

## Disclosure

The author declares no conflict of interest.
